# Advanced lung cancer patient with isolated heparin-induced thrombocytopenia: A case report

**DOI:** 10.1097/MD.0000000000029461

**Published:** 2022-07-15

**Authors:** Kai Yu, Hui Jiang, Li-hua Han, Zhi-ying Tong, Wan-min Wang, Yan Shang, Jia-yi Zhao

**Affiliations:** a Department of General Practice, Changhai Hospital, Naval Medical University, Shanghai, China; b Department of Pathology, Changhai Hospital, Naval Medical University, Shanghai, China; c Department of Laboratory, Changhai Hospital, Naval Medical University, Shanghai, China; d Department of General Practice, China Rongtong Medical Healthcare Group Co., Ltd., Shanghai, China.

**Keywords:** heparin-induced thrombocytopenia, rivaroxaban, lung cancer

## Abstract

**Rationale:**

Heparin-induced thrombocytopenia (HIT), a potentially devastating form of drug-induced thrombocytopenia, occurs in patients receiving heparin for thrombosis prevention or treatment. An isolated HIT is characterized by decreased platelet counts without thrombosis, which are atypical and difficult to clinically find.

**Symptoms and clinical findings:**

A 33-year-old female patient’s admission examination revealed elevated D-dimer levels. After prophylactic anticoagulation using low-molecular weight heparin, her blood platelet counts were rapidly decreased, whereas her D-dimer levels increased, followed by presentations of chest tightness, abdominal pain, and skin itching without thrombosis. After excluding all the other causes of thrombocytopenia, HIT was suspected. Her 4Ts score was 5 points, and enzyme-linked immunoassay for platelet factor 4 (PF4)/heparin antibodies was positive, indicating isolated HIT.

**Diagnoses, interventions, and outcomes:**

The patient was diagnosed with advanced lung cancer presenting with isolated HIT. We immediately stopped low-molecular weight heparin and initiated rivaroxaban for anticoagulation. We administered thrombopoietin (TPO) and avatripopal maleate tablets to increase blood platelet counts, whereas intravenous immunoglobulin (IVIG) was administered to stimulate her immune system. The patient’s thrombocytopenia was successfully treated without thrombosis and bleeding complications.

**Lessons:**

Rivaroxaban is a potential option for tumor preventive anticoagulation and HIT treatment. Early HIT identification is necessary. After identification, the 4Ts score as well as PF4/heparin antibodies should be assessed and appropriate anticoagulants selected based on patients’ conditions.

## 1. Introduction

Heparin-induced thrombocytopenia (HIT) is a rare drug-induced immune-mediated disease. In susceptible patients, heparin treatment generates antibodies against the heparin-PF4 complex, leading to platelet activation and aggregation.^[1]^ For heparin-exposed patients, HIT incidences range from 0.5% to 5% with a mortality rate of >20%.^[2]^ The most common clinical symptom of HIT is the marked decrease in platelet counts with thrombosis (HITT) or without thrombosis (isolated HIT). A few patients may present acute systemic reactions; however, HIT-related bleeding is rare.^[3]^

## 2. Case report

**Figure 1.**
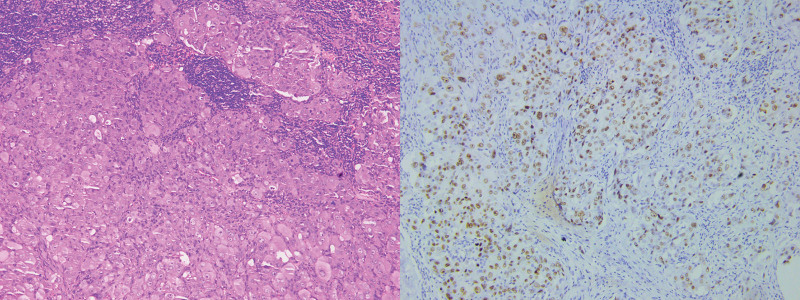
Hematoxylin and eosin staining of biopsy tissues of the left neck lymph nodes confirmed metastatic adenocarcinoma (100× magnification).

**Figure 2.**
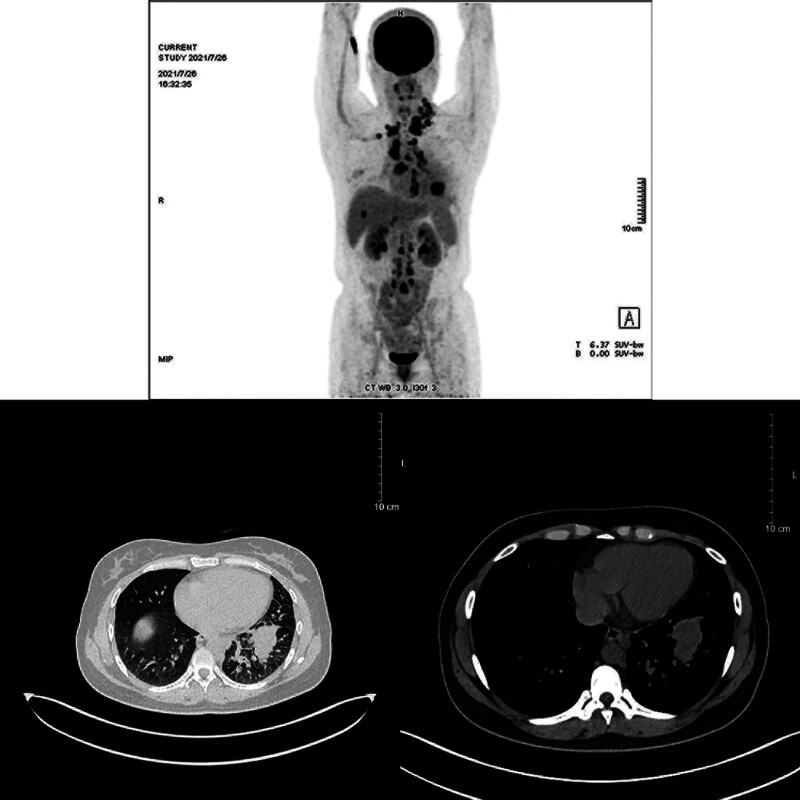
Imaging of PET/CT. Irregular soft tissue mass in the basal segment of the lower lobe of the left lung, considering peripheral carcinoma. Left hilum, mediastinum, left deep cervical space, left posterior cervical triangle, bilateral supraclavicular fossa, bilateral posterior space of the diaphragmatic angle, and retroperitoneum multiple lymph node metastases. Metastatic carcinoma is in the right anterior lobe of the liver. PET/CT = positron emission tomography/computed tomography.

A 33-year-old female patient was admitted to our hospital on July 30, 2021. The patient had continuously coughed and expectorated for 2 months and presented with a 10-day left neck mass. Before admission, the neck mass was examined for puncture pathology. Immunohistochemical staining showed metastatic carcinoma of the left neck lymph node (Fig. 1), whereas positron emission tomography/computed tomography (PET/CT) (Fig. 2) revealed that the peripheral carcinoma of the lower lobe of the left lung was accompanied by metastasis of the right anterior lobe of the liver and multiple lymph node metastasis. The diagnosis was adenocarcinoma of the lower lobe of the left lung T4N3M1c (liver, supraclavicular lymph nodes, and retroperitoneum lymph nodes) IV-B PS1, ROS1 (+), EGFR (−), ALK (−). She had an abnormal blood coagulation, a cesarean section in 2014 and an abortion in 2017 (a scarred pregnancy). She had a 10-year history of smoking, about 20 cigarettes per day. Three months before admission, the patient had used steroid hormone medications to control body weight, however, after presenting chest tightness and palpitation symptoms, the patient stopped the medications. In addition, her mother also had an abnormal blood coagulation.

**Figure 3.**
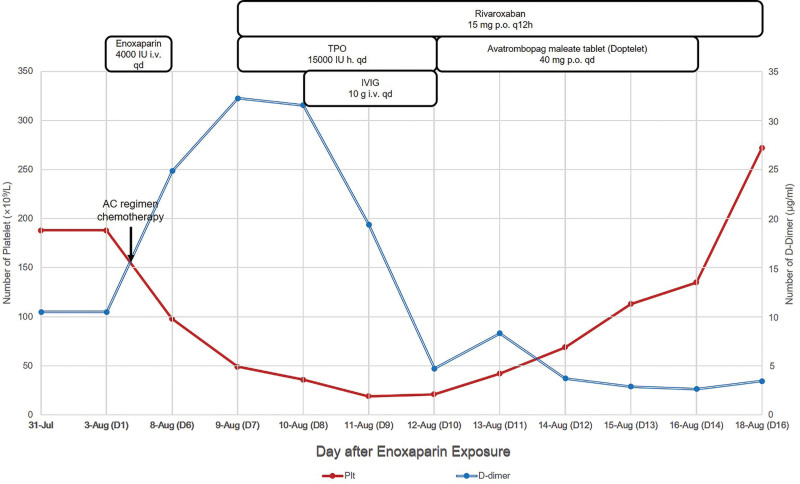
Time course changes in platelet and D-dimer counts. After enoxaparin treatment, platelet counts decreased and were lowest on day 9, then, enoxaparin was stopped and replaced with rivaroxaban. Gradually, platelet counts rose to normal levels. D-dimer levels peaked on day 7, rapidly declined after rivaroxaban treatment, and remained at low levels after day 12.

After admission, her platelet count was 188 × 10^9^/L, D-dimer levels were 10.48 μg/mL (Fig. 3), whereas C-reactive protein (CRP) concentrations were 14.6 mg/mL. On August 3, there was slight redness and pain around the catheterization of the right upper arm vein. Deep vein ultrasound of the 4 limbs revealed flow patency in the bilateral axillary vein, iliac vein, and femoral vein. Due to the hypercoagulable state of the tumor, enoxaparin 4000 IU q.d. was used for prophylactic anticoagulation. Since a genetic test report was not available, AC regimen chemotherapy (pemetrexed 800 mg D1 + carboplatin 400 mg intravenous D1) was administered on August 5. On August 6, there were intermittent pruritus of both lower legs and right upper arm with chest tightness and slight left upper abdominal pain. On the night of August 7, she complained of skin itchiness and needled finger. A few moments later, she felt a distending left upper abdomen, had cold sweats, was slightly nauseated and uncomfortable. On August 8, her platelet counts dropped to 66 × 10^9^/L, D-dimer levels increased to 24.85 μg/mL, while her CRP concentrations reduced to 7.94 mg/mL. In the next few days, there was a progressive decline in her condition. On August 10, cTnI concentrations were 5.44 ng/mL, electrocardiogram (ECG) revealed II, III, and an inverted aVF’s T-wave, which necessitated consultations with cardiology, pulmonary, hematology, and oncology physicians. Platelet, plasma, and cryoprecipitates were prepared. Assessments of her platelet specific and tissue-associated fusion antibodies were negative. Then, bone marrow aspiration was performed. Bone marrow morphology revealed mild morbid hematopoiesis of the granulocytic and erythroid lineages, marked megakaryocytic hyperplasia with a left shift of the maturation curve. Thrombotic thrombocytopenic purpura, immunologic thrombocytopenic purpura, myelosuppression after chemotherapy, and lung cancer with bone metastases were temporarily excluded. After 6 days of enoxaparin injection, the patient’s platelet counts started to decline, and the minimum count was 18 × 10^9^/L. Platelet counts decreased by 90.4%, while the 4Ts score was 5 points, thus, a moderate clinical possibility of HIT was considered. Anticoagulation with enoxaparin 4000 IU i.v. qd was changed to rivaroxaban 15 mg p.o. q12h. During this period, she was administered with thrombopoietin (TPO) 15,000 IU h. qd (August 9–12) and avatrombopag maleate tablet (Doptelet) 40 mg p.o. qd (August 12–16) to increase the platelet counts. Moreover, intravenous immunoglobulin (IVIG) 10 g i.v. qd was administered (August 10–12) to stimulate her immune system. On August 18, the patient had no discomforts such as itching, chest tightness, and abdominal pain. Her platelet counts increased to 272 × 10^9^/L, D-dimer levels dropped to 3.45 μg/mL while her CRP levels increased to 28.77 mg/mL. Deep vein ultrasound showed bilateral iliac and a smooth femoral venous flow, while heart ultrasound did not reveal any significant abnormalities. Enzyme-linked immunoassay for platelet factor 4 (PF4)/heparin antibodies (Fusheng Industrial Co., Ltd, Jinshan District, Shanghai, China) was positive. Therefore, her diagnosis was classical HIT without thrombosis (isolated HIT). Anticoagulation therapy with rivaroxaban was continued after discharge.

## 3. Discussion

Due to lung cancer, our patient was hypercoagulable and developed HIT during anticoagulation with enoxaparin. Thrombocytopenia often occurs after anticoagulant therapy with unfractionated heparin (UFH) or low-molecular weight heparin (LMWH) for prophylactic deep vein thrombosis in hospitalized patients with malignancies, thereby posing a challenge in HIT diagnosis. Previous guidelines recommend using LMWH or warfarin to prevent thrombosis during treatment. However, the best prevention for cancer-related venous thromboembolism has not been conclusively established. Various studies have reported that oral anticoagulants, such as rivaroxaban are favorable prevention and treatment options.^[4]^ The American Society of Clinical Oncology and the Spanish Society of Clinical Oncology (SEOM) updated their clinical practice guidelines with the introduction of oral anticoagulants as prevention and treatment options for thrombosis in malignant tumor patients.^[5,6]^ The administration of rivaroxaban in some high-risk patients, including patients with tubular gastrointestinal cancer, cancer patients at risk of bleeding from reproductive urinary tract, bladder, or nephrostomy, or patients with active gastrointestinal mucosal abnormalities such as duodenal ulcers, gastritis, gastroenteritis, or colitis, may increase the risk of bleeding.^[7]^ Therefore, the importance of individualized treatment cannot be stressed further.

When the patient had chest tightness, abdominal pain, skin pruritus, and other symptoms, accompanied by a sharp decline in blood platelet levels, combined with the patient’s history of LMWH treatment, we quickly started the 4Ts score and evaluated for the presence of the PF4/heparin antibody. The American College of Clinical Pharmacy guidelines, UK HIT guidelines, and expert consensus on HIT of China in 2018 have provided the guidelines for HIT diagnosis and treatment. The platelet function tests, such as serotonin release assay and heparin-induced platelet aggregation assay (HIPA), are second-line diagnostic approaches. The 4Ts score system should be used to determine the probability, confirm and expand the diagnosis by antibody testing. Clinically, enzyme-linked immunosorbent assay (ELISA), which is convenient, easy to perform, time-consuming, and highly sensitive, but with a limited specificity, is the mainstay of HIT antibody testing.^[1]^ Currently, ELISA is widely used as the negative-discharge test. Its clinical applications should be combined with the 4Ts score to identify HIT patients as early as possible, reduce complications as well as death and disability risks. Therefore, it is necessary to strengthen the HIT antibody screening to improve on its diagnostic rate.

Multiple anticoagulant drugs are available for anticoagulant treatment of HIT. The FDA-approved drugs include bivalirudin and argatroban. The American Society of Hematology guidelines recommend argatroban or bivalirudin for patients who are critically ill, have an increased risk of bleeding, or may require urgent treatment. In cases of an indication for continued anticoagulation, rivaroxaban may be preferred for patients with acute isolated HIT. Bivalirudin and argatroban were parenterally administered, required monitoring of coagulation indicators such as activated partial thromboplastin time, were expensive, and were associated with high levels. After comprehensive considerations, rivaroxaban, a direct oral anticoagulant drug (DOAC), was administered. DOACs are widely used for the prevention and treatment of venous thromboembolism; however, it has not been established whether they can be used to treat HIT. DOACs are brief, do not require repeated testing of thrombus and coagulation functions, and do not cross react with PF4/heparin antibodies. In the past 5 years, the applications of DOACs for HIT treatment is gradually increasing,^[8–10]^ making it a potential alternative to the conventional therapeutics. Among them, Rivaroxaban is the most available, and it can attenuate further platelet reduction after HIT as well as reduce thrombotic events. This indicates that DOACs are efficacious when applied in HIT. Based on the available studies, DOACs have a promising future in HIT therapy; however, large-scale randomized controlled clinical studies on DOACs should be performed to provide a reliable basis for treatment.

In conclusion, rivaroxaban is a better choice for preventing thrombosis in many tumor patients. According to American Society of Hematology guidelines, it is also recommended as the first choice for patients with acute isolated HIT. After given venous catheterization with heparin tube sealing, patients present with redness and pain of the skin, or symptoms such as chest tightness, abdominal pain, and skin itching in response to the use of heparin. The possibility of HIT should be determined after exclusion of thrombocytopenia and other causes affecting platelets, coagulation, or thrombosis. 4Ts score should be given as soon as possible. Patients with a moderate to high-clinical likelihood of 4Ts score should undergo PF4/heparin antibodies as soon as possible to confirm the diagnosis. For these patients, heparin should be immediately stopped, and appropriate anticoagulant drugs such as rivaroxaban selected based on patients’ conditions.

### Author contributions

Supervision: Yan Shang, Jia-yi Zhao.

Writing—original draft: Kai Yu.

Writing—review and editing: Kai Yu, Hui Jiang, Li-hua Han.

Data curation: Zhi-ying Tong, Wan-min Wang.

Visualization: Hui Jiang.
